# Sustainability of healthcare improvement: what can we learn from learning theory?

**DOI:** 10.1186/1472-6963-12-235

**Published:** 2012-08-03

**Authors:** Einar Hovlid, Oddbjørn Bukve, Kjell Haug, Aslak Bjarne Aslaksen, Christian von Plessen

**Affiliations:** 1 Institute of Social Science, Sogn og Fjordane University College, Postbox 133, 6851, Sogndal, Norway; 2 Department of Public Health and Primary Health Care, University of Bergen, Bergen, Norway; 3 Department of Radiology, Haukeland University Hospital, Bergen, Norway; 4 Institute of Surgical sciences, University of Bergen, Bergen, Norway; 5 Department of Thoracic Medicine & Infectious Disease, Hillerød Hospital, Hillerød, Denmark; 6 Institute of Medicine, Faculty of Medicine and Dentistry, University of Bergen, Bergen, Norway; 7 Department of Health Studies, Faculty of Social Sciences, University of Stavanger, Stavanger, Norway

**Keywords:** Quality improvement, Organizational learning, Learning theory, Sustainability

## Abstract

**Background:**

Changes that improve the quality of health care should be sustained. Falling back to old, unsatisfactory ways of working is a waste of resources and can in the worst case increase resistance to later initiatives to improve care. Quality improvement relies on changing the clinical system yet factors that influence the sustainability of quality improvements are poorly understood. Theoretical frameworks can guide further research on the sustainability of quality improvements. Theories of organizational learning have contributed to a better understanding of organizational change in other contexts. To identify factors contributing to sustainability of improvements, we use learning theory to explore a case that had displayed sustained improvement.

**Methods:**

Førde Hospital redesigned the pathway for elective surgery and achieved sustained reduction of cancellation rates. We used a qualitative case study design informed by theory to explore factors that contributed to sustain the improvements at Førde Hospital. The model Evidence in the Learning Organization describes how organizational learning contributes to change in healthcare institutions. This model constituted the framework for data collection and analysis. We interviewed a strategic sample of 20 employees. The in-depth interviews covered themes identified through our theoretical framework. Through a process of coding and condensing, we identified common themes that were interpreted in relation to our theoretical framework.

**Results:**

Clinicians and leaders shared information about their everyday work and related this knowledge to how the entire clinical pathway could be improved. In this way they developed a revised and deeper understanding of their clinical system and its interdependencies. They became increasingly aware of how different elements needed to interact to enhance the performance and how their own efforts could contribute.

**Conclusions:**

The improved understanding of the clinical system represented a change in mental models of employees that influenced how the organization changed its performance. By applying the framework of organizational learning, we learned that changes originating from a new mental model represent double-loop learning. In double-loop learning, deeper system properties are changed, and consequently changes are more likely to be sustained.

## Background

Quality improvements in health care that are not sustained are a waste of resources. Falling back to old, unsatisfactory ways of working can be frustrating and increase the resistance to later initiatives to improve care. Few publications have reported sustainability of healthcare improvements. In a systematic review, the median follow-up time for interventions that sought to improve the quality of care was less than 1 year [1]. Consequently, little is known about the factors that contribute to the sustainability of improvements [2,3]. This makes such research sorely needed [4].

Over the past few decades, an understanding of healthcare quality as a system property has emerged [5-7]. Accordingly, the quality of health care primarily depends on the function of the system and to a lesser degree on the skills of individuals [5]. Changing the system is therefore the most effective route to improvement; i.e., an organization needs to change its way of operating to produce improved outcomes, and these changes must be maintained to sustain the improvements [5,8].

The sustainability of systemic change is poorly understood. Use of theoretical frameworks allows for an understanding of factors that contribute to sustainability [9,10]. In particular, theories of organizational learning explain crucial aspects of change in organizational behavior. Argyris and Schön [11] defined learning as the translation of new knowledge to altered behavior that is replicable. Quality improvement implies that an organization needs to alter its behavior, and that the behavioral changes must be replicable to sustain improvements. Sustained improvement after systemic change can thus represent a case of organizational learning. A framework of organizational learning can be used to explore the factors that influence the sustainability of improvements.

In this article, we use an organizational learning framework to explore a case that demonstrated sustained improvement. The case is Førde Hospital’s project redesigning their pathway for elective surgery to reduce cancellations. Reasons for cancellations are complex and related to patients, organizational issues, and clinical staff [12,13]. Cancellations are caused by a clinical system performing sub-optimally, e.g., poor scheduling, inadequate medical pre-assessment, and facility shortcomings [14-19]. Reducing cancellations requires changes in the clinical system. Changing the clinical system requires organizational change through organizational learning [5]. Organizational learning becomes manifest through new organizational routines, and the effects of these new routines can be measured [20,21]. Organizational learning in our case should therefore be reflected in reduced cancellation rates. We have previously demonstrated how the redesign of the surgical pathway at Førde Hospital caused a significant reduction in cancellation rates from 8.5 % to 4.7 % that was sustained over 2 years [22]. The case is thus an example of sustained improvement through organizational learning, and it should be suitable for exploring factors contributing to sustain the improvements.

## Methods

### Context

Førde Hospital is a district general hospital in a small town in Norway, population 10,000. The hospital has 7 operating suites and 34 surgical beds. Like most hospitals in Norway, Førde Hospital is publicly owned and financed. The local health authority also includes two smaller local hospitals. Altogether, the three hospitals serve a population of approximately 107,000. All patients have full healthcare coverage through the national state insurance.

The cancellation of planned surgeries is a known problem in health care that affects patients, diminishes quality of care, wastes resources, and increases healthcare costs. Complaints from patients and high cancellation rates indicated that the pathway for elective surgery was not optimal at Førde Hospital, and the hospital therefore set out to redesign the entire pathway. The project involved the surgical departments at the hospital (ophthalmology, general surgery, gynecology, orthopedics, and ear, nose, and throat). Altogether 280 full-time equivalents work in these departments.

Four different project groups with a total of 40 employees were formed. Each group was given a mandate to redesign parts of the pathway. The changes that were implemented included one common entry point for all referrals, earlier clinical patient assessment, improved information flow among staff members, patient participation in selecting the date for surgery, and improved coordination and scheduling of operations.

### Theoretical framework

The Evidence in the Learning Organization (ELO) model describes how healthcare organizations *learn, create, and share knowledge about evidence-based practices and the system issues that facilitate or inhibit these learning processes*[23] Therefore, we found that this model is appropriate as a framework for our analysis. The model is based on four main themes: *inquiringdecidingrelating*, and *interpreting*. Together, these themes *represent the process required for organizations to learn and share new knowledge more effectively*[23]:

1. Inquiring: Are members ready to inquire on behalf of teams/organizations to facilitate the loop learning processes?

a. Acquiring: Do they possess technical skills related to locating resources and communicating feedback about this inquiry (e.g., Information Technology training)?

b. Informing: Do they possess the cognitive skills (i.e., EBM skills) that support evidence-based decisions?

c. Transforming: Do they possess cognitive traits that facilitate behaviors for inquiry (e.g., internal learning motivation)?

2. Deciding: Are members and teams utilizing effective decision processes to integrate evidence into healthcare decisions?

a. Deliberating: Are they comparing and analyzing new working goals/strategies and structures/processes that will lead to better decisions (e.g., weighing alternative work procedures)?

b. Decision-taking: Are they using appropriate decision methods/tools to support better decision-making (e.g., computer-assisted decision tools)?

c. Evaluating: Are they using adequate analytical methods (qualitative or quantitative) to measure outcomes of evidence-based decisions (e.g., adequate audit and feedback)?

3. Relating: Are members, teams, and organizations facilitating evidence-based practices through effective organizational communication and relationships?

a. Sharing: Do the organizational communication structures and processes facilitate sharing knowledge (e.g., adequate information networks)?

b. Cooperating: Are teams available and functioning to facilitate efficient knowledge generation and evaluation (e.g., team composition and roles)?

c. Advocating: Is there adequate and sufficient leadership with effective motivational strategies to induce organizational cultural change toward learning (e.g., incentives, championing, leadership style, etc.)?

4. Interpreting: Are members and teams sensing the need for evidence-based practice innovations and explicitly describing their tacit knowledge?

a. Judging: Are they properly evaluating judgments about the outcomes of decisions and needed practice changes (i.e., testing for epistemic gaps)?

b. Knowing: Are they building new models of shared understanding based on the results of evidence-based decision-making (i.e., interpreting/integrating with communities of practice)?

c. Formulating: Are they codifying this new knowledge (e.g., team-tested practice recommendations) for organizational consumption?

The ELO model itself does not specifically elaborate on how new knowledge is created, how individual learning is transformed to organizational learning, or what organizational mechanisms are involved in the change process. These questions are important for organizational learning. To better understand these processes, we included four of the theoretical frameworks that underlie the ELO model: Argyris’ [24] loop learning, Kim’s [25] concept of organizational learning, Nonaka’s [26]*Socialization, Externalization, Combination, and Internalization* (SECI) model, and the framework of complex adaptive systems (CAS) [27].

Together these frameworks help explore factors that sustain organizational changes. The concept of single- and double-loop learning explains the actual learning process in the organization [24]. Kim’s [25] model explains the transformation from individual to organizational learning through mental models. The SECI model sheds light on how these mental models are incorporated and shared at the organizational level. The framework of CAS elucidates consequences interdependencies in a clinical system can have for organizational behavior and performance [28].

### Loop learning

The concept of single- and double-loop learning explains the actual learning process in the ELO model [24]. In single-loop learning, a defect or mismatch between expected and observed outcomes is corrected, leaving the underlying theory for the action unchanged [24]. The feedback loop from the actual experience does not change the basic assumptions or decision-making rules that govern the action that corrected the defect. In double-loop learning, the detected defect is corrected, and the feedback loop from what is experienced during this process also changes the underlying theory or decision-making rules of the action that corrected the defect.

Double-loop learning can occur in organizations *when individuals inquire on behalf of the organization in such a way as to lead to change in the values of the organizational theory in use*[24]. In contrast to single-loop learning, double-loop learning changes the individuals’ understanding of the fundamental *theories* and *values* that guide organizational behavior [24]. Double-loop learning is thus a deeper change than single loop-learning because it changes the underlying system that produces the current organizational behavior.

### Organizational learning through changed mental models

Earlier models of organizational learning did not describe how individual members of an organization learn or how the organization learns. According to Kim [25], shared mental models can be viewed as the link between individual and organizational learning. *The cycles of individual learning affect learning at the organizational level through their influence on the organization’s shared mental models.* When referring to mental models, Kim used Senge’s [29] definition: *deeply held internal images of how the world works, which have a powerful influence on what we do because they also affect what we see.*

Kim’s 1993 model also incorporates Argyris and Schön’s concept of double-loop learning. *Organizational double-loop learning occurs when individual mental models become incorporated into the organization through shared mental models, which can then affect organizational action*[25]. Argyris and Schön define organizational learning as the way people jointly construct maps [11]. These maps can be viewed as the mental models that are shared by the organization and compatible with the concept of the organizational theory in use [24].

### The SECI model

Individual mental models can be viewed as tacit knowledge. According to Kim, making this tacit knowledge explicit is crucial to the development of new shared mental models in an organization [25]. The SECI model [26] explains how organizations dynamically share, create, and maintain knowledge. Knowledge is created through interactions between tacit and explicit knowledge through four modes of knowledge conversation: *socialization, externalization, combination, and internalization*[30]. Thus, the SECI model can help understand how individual mental models can be turned into shared mental models in the organization because it explains how tacit knowledge can be made explicit and shared in an organization. The model has been extended by introducing the concept of *ba*[30]. *Ba* can be defined as *a shared context in which knowledge is shared, created, and utilized*[30]. This shared context is important because it affects what kind of knowledge is shared and how the knowledge that is created is utilized.

### Complex adaptive systems

In line with the increasing complexity of healthcare services, traditional organizational models have exhibited shortcomings, especially in explaining how change occurs. New ways of looking at healthcare organizations have evolved. The literature has advocated use of the CAS framework to explore change processes in health care [31-33]. As opposed to traditional organizational models, CAS explores organizational change by directing attention to the interdependency of the different organizational elements and not towards the elements themselves [28]. CAS can thus help us understand the importance of the relationships and patterns of actions between individuals in a clinical system [34]. These relationships are dynamic, non-linear, and evolve with time. Small changes in one part of the system can lead to huge consequences in a different part [34]. Changes in mental models at an organizational level may influence the relationships and patterns of actions between individuals in the clinical system. We used CAS to better understand the connection between clinicians’ change in mental models and their revised understanding of interdependencies in the clinical system, and how this new understanding affected organizational behavior.

### Design

Given the scarce knowledge on sustainability of healthcare improvements, the character of our study is explorative. Thus we used a qualitative case study design grounded in the theoretical framework of learning theory [35]. Our case is the redesign of the clinical pathway for elective surgery at Førde Hospital.

### Data collection

We used purposive sampling to explore the organizational changes at the hospital [36]. Our focus was on organizational rather than the individual perspectives. Thus we included informants with different roles in the hospital. With assistance from the hospital administration, we recruited informants with different professional backgrounds and work experience as well as varying degrees of involvement in the improvement project.

Case study evidence initially consisted of administrative documents. These documents described the overall aim of the improvement project and the mandate of the improvement groups at Førde Hospital. We did not conduct a formal document analysis, but the documents provided us with background information for the interviews.

One of the authors (EH) conducted all interviews during June and July 2010. Seventeen of the interviews were conducted face to face, and three on the telephone. The length of the interviews varied between 20 to 70 minutes. Each respondent was interviewed once. EH wrote case notes for each interview. Our purpose was to maximize information [37,38]. Based on the case notes, the last few interviews did not add any new substantial information. Thus, we reached redundancy, and our sample size was sufficient for the purposes of the study [37,38].

EH conducted in-depth interviews. Based on our theoretical framework, he asked questions having to do with the following themes: identification of a need to change, planning the change, actions taken to induce change, outcomes of change, and adaptations of interventions. He used open-ended questions, e.g., How did the interviewees realize that they needed to change? What did they do to induce changes? How did this change affect their work? At the beginning of the interviews he collected demographic data (gender, profession, degree of involvement in improvement work, leader responsibilities, and years of work experience at the hospital). We grouped the degree of involvement in improvement work in employees who participated in the project groups, i.e., those directly involved in planning and execution, and employees who did not participate in the project groups but whose daily work was affected by the changes.

### Analysis

**Figure 1:**
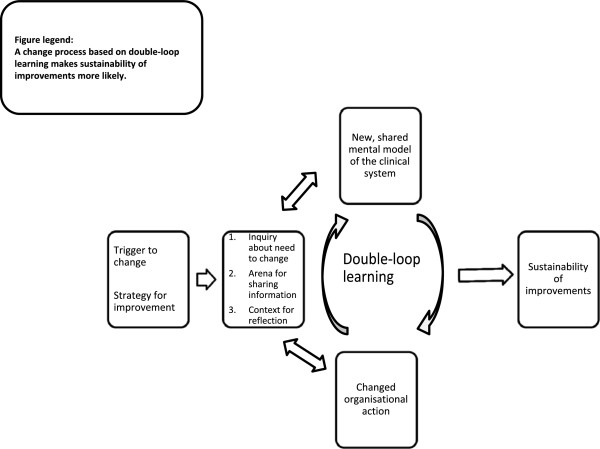
Factors that contribute to sustained improvements.

We analyzed the interviews in the three steps described by Creswell [39]: preparing and organizing, reducing into themes through a process of coding and condensing, and representing in a figure and a discussion. We taped the interviews, transcribed them verbatim, and transferred them to HyperRESEARCH 2.8.3 computer software (Research Ware, 2009) for coding. We developed an initial coding scheme based on the main themes in the ELO model. EH coded the entire data set. During the analysis new codes were added based on the data [39]. Through an iterative process of coding, reflecting on the codes, and condensing, we identified common themes [37]. We interpreted the themes with regard to our theoretical frameworks and represented the relationship between the themes in Figure 1[39,40].

As recommended by Barbour, [41] the other authors validated excerpts of the data set to validate the coding and the quotations that we present to illustrate our findings. To enhance the rigor of our analysis, three key respondents validated a narrative of how interventions were planned and implemented in the hospital [38,42]. We adapted the quotations, without changing the meaning, to enhance readability and maintain confidentiality [40]. A professional translator translated the quotations presented in this article into written English.

### Ethical considerations

The Western Department of the Regional Committee for Medical and Health Research Ethics in Norway deemed a full ethical review unnecessary because the study did not use sensitive patient data. The study protocol was accepted by the Norwegian Social Science Data Services, which reviewed ethical aspects related to collecting and handling data (voluntary participation based on informed consent, anonymity of informants, and presence of appropriate data storage protocols).

## Results

We interviewed 20 employees with different professional backgrounds and varying degrees of involvement in the improvement work. Characteristics of the interviewees are provided in Table 1.

**Table 1:** Characteristics of the interviewees

**Professional group**	**Number of informants**	**Participated in improvement groups**	**Not directly engaged in improvement work**	**Leaders**	**Gender (female/male)**	**Years of work experience at Hospital <5 5–10 >10**		
**Physician**	9	4	5	4	1/8	4	1	4
**Nurse**	7	5	2	3	5/2	1	3	3
**Secretary**	2	0	2	1	2/0		1	1
**Administrators (project support)**	2	2	0	1	2/0	1	1	
**Total number**	20	11	9	9	20	6	6	8

We structure the presentation of our findings around the four main themes of the ELO model. Where relevant, we present representative quotations from the interviews to illustrate our findings.

### Inquiring

Complaints from patients and high cancellation rates indicated that the pathway for elective surgery was not optimal. At the beginning of the project, there was a unified understanding that the pathway needed to be improved. Four multidisciplinary project groups were formed to suggest a redesign of different parts of the pathway. The inquiry was initiated in these groups, and clinicians outside the groups were involved through regular meetings and dialogue.

Within the organization, there was a desire to try to improve the flow of patients.

Middle manager in project group.

There were lots of information meetings along the way. People were supposed to make suggestions; they could write suggestions on pieces of paper, which were posted on the wall. They could say what they thought about the various stages in the process.

Physician not in project group.

The health authority had developed a common platform and strategy for conducting improvement projects. The focus was on detecting systemic problems through equally addressing the four perspectives: professional, patient, and management quality (resource utilization), and staff satisfaction. The balanced approach among these four quality dimensions contributed to making the project understandable and increased acceptance among frontline clinicians.

The project groups mapped the current state of the clinical system and then began inquiring about how the system ought to work in the future and what changes needed to be made. Necessary process data were extracted from the patient administrative system. The project groups received guidance about improvement techniques. They used simple tools, such as Post-It Notes, to visualize the clinical pathway.

You may have really good project support, but if you don’t have really good ideas, good staff, creative staff, then all you’ll get are minor adjustments or copies of what others do.

Staff member offering support in project group.

Cases of patients were used in the inquiry process to emphasize the patient experience in the pathway. Moreover, the team used ideas from a site visit to a hospital that was considered to have a better practice. Interventions suggested in the literature were also used. The inquiry was a stepwise process influenced by activities in the *relating* and *interpreting* phases of the model. New knowledge created in these phases revealed new areas of inquiry.

### Deciding

The project combined top-down and bottom-up approaches. The improvement strategy secured a sound foundation with the top management, whereas the frontline professionals were left with sufficient room to find new ways to redesign their own work processes. The project groups suggested interventions and tried to build a consensus for their suggestions through the involvement of and dialogue with clinicians outside the groups. Staff from the support unit provided the groups with structure and process data from the patient administrative system that served as the groundwork for their decisions.

Middle managers in the project groups mostly decided themselves which interventions to implement. Interventions in the project were in accordance with published evidence (e.g., earlier patient assessment, involvement of patient in decisions for scheduling operations, and calling the patient 2 days prior to surgery).

Those with expertise in project and improvement measures took part throughout the preliminary investigation stage and knew exactly what had been done before, what had been decided, and what the plans for the future were.

Middle manager in project group.

Additionally, the *deciding* phase was a stepwise process influenced by the new knowledge created in the *relating* and *interpreting* phases of the model. This new knowledge provided a new perspective and new areas of inquiry, which again could lead to new decisions. The middle managers participated in the actual clinical processes that were affected by the interventions, thereby instantly learning about the effects of their decisions. This feedback was considered more valuable than measurements such as cancellation rates because it was direct and without delay. Sometimes this feedback revealed a need to revise previous decisions.

Implementation was time-consuming and difficult because of resistance in the organization. Consistent follow-up by middle managers over an extended time was necessary to actually implement the decisions that were made. Through their participation in daily work, middle managers exemplified the new ways of working and demonstrated the importance of following new routines.

I’ve learned that involving the relevant staff is not enough. Unfortunately, we need those enthusiasts, too. This has not been a success only because of involvement, Post-It Notes, and conclusions. If that were the case, we would not have progressed a single step. And that is something I think that improvement theorists need to take more seriously: that is, that the project itself is only one per cent, or ten per cent. Ninety per cent is the consistent follow-up. And that is generally extremely unpleasant.

Middle manager in project group.

### Relating

The meetings in the project groups were the most important arena for sharing and reflecting on information. The strategy of the top management was to include all of the professional groups that participated in the work process; thus, they convened interdisciplinary project groups. In these groups, frontline clinicians shared information, reflected on it, and related it to their own work and the clinical pathway as one whole to detect areas for improvement. Through this process, tacit knowledge was made explicit and shared.

Reflection and communication was not confined to the project groups. Through the active involvement of clinicians outside the project groups, new knowledge was spread and shared throughout the organization.

You got to sit in a group with the doctor, the nurse, the director, and the porter and look at all the problems. It’s not only about my challenges in dealing with a patient scheduled for an operation in an hour. There is actually an entire surrounding complex that has to work.

Middle manager in project group.

### Interpreting

In accordance with the improvement strategy, the reflections by the project groups were set in the context of how the entire pathway could be improved. Considering this context enabled a new understanding of the clinical system to emerge. Clinicians realized that their former work processes had been fragmented and that they had lacked an understanding of how the hospital worked as a whole. Gradually, the focus shifted from their small, familiar part of the patient flow to how all of the various elements needed to interact to improve overall system performance.

Furthermore, individual clinicians reflected on how their own work contributed to the pathway and began to realize how dependent they were on each other and how crucial everybody’s contribution was for an optimal pathway. Through this reflection, the organization improved its understanding of the clinical system and its interdependencies.

You see more than your own little task, and you see how you can become a bottleneck for others’ tasks without even knowing it. I think that getting to see the whole process and to see that you actually are one link in a long chain helps people to see things more holistically.

Staff member offering support in project group.

The way it used to be, in many areas the big picture fell apart; work was so fragmented.

Physician in project group.

Each separate section had its own books with patients needing surgery, and everybody tried to plan their operation schedules on the basis of these. But there was no coordination; nothing brought things together in terms of the resources available on the ward as a whole.

Nurse in project group.

The new understanding of the clinical system changed the mental model. Moreover, it was codified into altered individual and organizational behavior. New organizational routines were created and implemented, in which the new system perspective was taken into account. For example, before the intervention the different surgery departments managed their own scheduling of operations, with no coordination among the departments. As part of the intervention, a new computer application was introduced for scheduling surgery across all surgical departments. This made waiting lists and schedules transparent across departments. Furthermore, a capacity coordinator position was created that was empowered to coordinate planning across the departments. Scheduling surgery also became more dynamic because waiting lists were considered when slots for surgery were assigned among departments.

The most important thing, I think we’ve learned is that it was very easy to sit and just look at your own sphere when working out procedures and general standards for patient scheduling. When we all sat down together and tried to create something, it required a mental readjustment so that we had to think, “This isn’t just about my area; it also affects others.”

Physician in project group.

*We* [anesthesiology and surgery] *have probably become closer; yes, we have. It isn’t uncommon that we now are in touch at the early stages to discuss a patient’s medical problem. Then, together, we work out a plan for preparing for the operation.*

Physician not in project group.

The improved system awareness influenced the inquiry and decision processes. As staff members became aware of the complexity of their clinical processes and their understanding of the interdependencies of the various elements grew, they discovered new problems and possible solutions.

*When the head got a look at the clinic waiting lists, it became clear that there was a whole ocean of things that needed to be tackled. And these are things that we didn’t know about before, because the system hadn’t been transparent*.

Middle manager in project group.

Now we see the big picture with regard to the operation schedule, and this means that we now discover ahead of time if two patients are scheduled for procedures that require the same instruments and thus re-sterilization. This used to cause unnecessary waits.

Nurse in project group.

Clinicians were involved in modifying and adapting the interventions to the context. Their new understanding of the clinical system influenced how this was done. With the new understanding, effects of the interventions were evaluated and adapted according to how the entire clinical pathway was thought should work.

Then we had to have a discussion about what we meant by “urgent,” what kind of things really are urgent, and what kinds of things it makes no sense to mark as “urgent.” So it took some time to get that as good as it could be.

Secretary not in project group.

## Discussion

We begin by discussing our findings with regard to the theoretical framework and proceed to relate our findings to previous studies. We conclude with implications for quality improvement in health care.

### A new understanding of the clinical system and double-loop learning

By structuring and analyzing our data according to the four themes of the ELO model, we were able to construct a representation of how the learning process unfolded. Furthermore, our theoretical frameworks helped us identify factors that contributed to sustain the improvements.

Our findings demonstrate that employees at the hospital developed a revised and deeper understanding of their clinical system and its interdependencies during the course of the improvement project. This new understanding had implications for organizational behavior. We consider this a key finding because it indicates a change in clinicians’ mental model of their clinical system that influenced organizational action.

This new understanding emerged from a dynamic process in which clinicians shared information, reflected on it, and related it to their everyday work situation. Consistent with the extended SECI model, including *ba*[26,30], individual tacit knowledge was made explicit and interpreted in a new shared context. This shared context was provided by the hospital leadership. Specifically this context involved how the various elements of the clinical pathway needed to interact to enhance the performance of the clinical system as a whole. Through this process of sharing and reflection among individuals across professional groups and departments, the employees’ new model of the clinical system was transformed into a mental model that was shared by the organization [25]. As pointed out by Kim [25], a changed mental model that is shared at the organizational level can serve as a foundation for double-loop organizational learning if it affects organizational action [24].

Individuals in a system tend to focus on their immediate surroundings and pay less attention to the functioning of the clinical system as a whole [43,44]. We observed the same kind of behavior at Førde Hospital before the project started. During the project clinicians shared and reflected on information with regard to how the performance of the clinical system as a whole could be improved. In line with the CAS framework we observed that clinicians revised their understanding of the clinical system as they acquired a better understanding of its interdependencies [43]. Clinicians’ improved understanding of the interdependencies in the clinical system affected three important stages of the change process: inquiry about what to change, change of organizational routines, and adaptations of interventions to the context.

As clinicians gradually improved their understanding of the clinical system and its interdependencies, they became able to detect system problems they previously had been unaware of. Failures prone to transitions between clinical entities were revealed as these transitions were evaluated from a new perspective, i.e., the clinical system as a whole. Furthermore, the new understanding led to a deeper and more precise understanding of the underlying causes of the quality problems.

Organizational learning becomes manifest through new or modified organizational routines [20]. In our case, clinical practice was altered as a consequence of a new understanding of the clinical system and its interdependencies. At the individual and group levels, physicians began cooperating in a new way that benefited the patients. At an organizational level, to offer one example, the hospital improved the scheduling and coordination of surgery by doing this across departments as opposed to department-wise as was done before the project. The new routine contributed to reducing cancellations and increasing the number of operations performed. Furthermore, remarks by the physicians demonstrated that their better understanding of the system of care facilitated the development of new organizational procedures in general.

Frontline employees were engaged in suggesting adaptations and modifications of the interventions. The improved understanding of the system increased the employees’ awareness of the interaction between context and interventions and improved their ability to adapt interventions to specific situations. Moreover, the hospital increased the effectiveness of changes by fitting them to a constantly changing context in a way pointed out by Fixsen et al. [45].

Consistent with Berwick [5], improvements in our case were made by changing the clinical system. During this process, clinicians developed a deeper understanding of their clinical system and its interdependencies. This was transformed into a shared mental model at the organizational level. The shared mental model affected organizational action, indicating that double-loop organizational learning occurred [24]. According to our theoretical framework, organizational change that involves double-loop learning is more likely to be sustained because it alters the deeper, structural, and cultural properties of systems. The fact that the hospital was able to facilitate and induce systemic change through double-loop learning appeared to be important for understanding how improvements were sustained. In our case, important stages in the process of changing the system were based on double-loop learning: inquiry about the need to change, change of clinical practice, and adaptations of interventions.

### Our findings in relation to earlier studies

An understanding of an organization as a system is a prerequisite for organizational learning [29]. The performance of a system is far more dependent on how the elements work together than on how each element performs separately [46]. According to Batalden and Davidoff [47], knowledge about *processes* and *patterns* is a prerequisite for improving the performance of a clinical system (i.e., knowledge about how clinicians interact to deliver the actual care that patients need). However, many health professionals are *process illiterate*, partially owing to the challenges of recognizing and understanding causal implications of their actions in a system [44,48]. In our case, clinicians improved quality by focusing on interdependencies, i.e., the way clinicians cooperated in their clinical processes to deliver care. By doing so, clinicians’ understanding of the implications of their actions grew, deepening their understanding of the clinical system.

Previous studies indicated that organizational learning in health care is fragmented (i.e., consisting of many learning cycles that are not interconnected) [21,49,50]. Contrary to our findings, Tucker and Edmondson [51] found that single-loop learning was dominant when nurses learned from mistakes. They suggested that this type of learning may mask the underlying structural problems of the system that could have been detected and corrected by double-loop learning. In our case, the hospital leaders were able to address underlying systemic problems through the dynamic process of inquiry, information sharing, and reflection, thereby facilitating double-loop learning.

Consistent with earlier studies [6,52], we found that multidisciplinary teams of professionals, combined with knowledge about improvement, was an important success factor in our case study. The staff that supported the project groups helped to structure an arena for reflection and sharing information. Furthermore, their guidance and assistance in mapping and visualizing the clinical system, along with their role in keeping track of decisions, were important for maintaining a system perspective during the inquiry process.

Perseverance from middle managers, who led the implementation process through their clinical work, was a key driver in overcoming resistance and implementing change. Consistent with previous findings, middle managers built and demonstrated knowledge about the clinical system through their work and leadership, thereby facilitating the spread of the new mental model [53,54]. By doing so, they maintained double-loop learning at the organizational level.

### Implications for quality improvement in health care

We report here that clinicians revised their understanding of their clinical system and developed a new mental model. The mental model was then shared by the organization and influenced the inquiry process, clinical practice, and the way interventions were adapted. These steps are illustrated in Figure 1. The improvement strategy triggered clinicians to inquire about their system and opened an arena for information sharing and for relating these activities to the context of the whole clinical system. These combined activities improved clinicians’ understanding of their clinical system. The process was circular as the new understanding influenced the actions that had induced it.

Our case demonstrates that clinicians’ understanding of their clinical system can be improved, partially depending on how a project is planned and conducted. The hospital’s general strategy for improvement influenced how this new understanding emerged. A fundamental part of the strategy was to provide an arena and structured approach for sharing information and involving frontline professionals in the inquiry about systemic problems by equally addressing patient, professional, and administrative quality [55]. By providing clinicians with an arena for sharing information and a context for reflecting on the shared information, the leadership facilitated the process that led to a revised understanding of the clinical system. We therefore suggest that it may be possible to influence clinicians’ understanding of their clinical system by paying close attention to how improvement work is planned and conducted. Table 2 summarizes and suggests implications of our findings for quality improvement work in health care.

**Table 2:** Implications for quality improvement in health care

**Leadership action**	**Desired change for organization**
Create a multidisciplinary arena for sharing information Provide a system context for interpreting shared information Provide guidance to clinicians about improvement knowledge	Revised and deeper understanding of the clinical system that is shared by the organization
Design and implement new organizational routines based on the new understanding of the clinical system	Change the system based on double-loop organizational learning
Facilitate continuous information sharing and reflection	Spread the new mental model in the organization
Modify and adapt interventions based on the new understanding of the clinical system	Sustained improvement

### Limitations, relevance, and further research

The retrospective study design has inherent limitations such as information bias and confounding; thus, we cannot prove causality between interventions and outcomes. However, combining a retrospective design with a theory-driven analysis allowed us to learn from a successful case by exploring how and why the improvement efforts worked and were sustained [9,10,56]. Retrospective interview data may be influenced by what respondents remember and how they emphasize various parts of their experiences. In our case, the respondents independently described how the improvement process changed their understanding of the clinical system and their own roles in this system. This finding was consistent across professional groups, regardless of the degree of involvement in the improvement work, thus increasing the credibility and trustworthiness of our analyses [37]. The rigor of our analyses was also enhanced by our use of complementary theoretical perspectives [57].

Our study is based on a single case that cannot be directly generalized. However, lack of sustainability of healthcare interventions is a substantial and ubiquitous problem [58,59]. The literature suggests that an incomplete understanding of the clinical system in not unique to our case; our findings are consistent with the literature and previous empirical findings [47,50]. In line with recommendations in the literature, we used theoretical perspectives to generate a middle-range theory, or context-dependent theory, which describes how clinicians’ increased understanding of their clinical system contributes to sustainability [9,10,60]. Despite the inherent limitations of a retrospective case study, we suggest that our theory may help hospitals to increase the sustainability of improvements.

Our study may also open a new line of research into sustainability of improvements. Future studies should address factors that improve individuals’ understanding of clinical systems, changes of mental models, sharing of mental models, and how these models affect organizational behavior [61]. A better understanding of these factors might eventually increase the sustainability of healthcare improvements.

## Conclusion

Our case study demonstrated that the clinicians developed a new understanding of their clinical system and its interdependencies. We suggest that the management can facilitate this kind of change by focusing on how frontline clinicians are involved in sharing and reflecting on information with regard to how the clinical system as a whole can be improved. The new understanding of the clinical system represented a change in mental models of employees that influenced how the organization changed its performance. Changes originating from a new mental model represent double-loop learning. In double-loop learning, deeper system properties are changed, and consequently improvements are more likely to be sustained.

## Competing interests

The authors have no competing interests.

## Authors’ contributions

EH designed the study, collected the data, analyzed the data and drafted the manuscript. OB participated in the design of the study, the analysis of data and helped to draft the manuscript. KH participated in the design of the study and helped to draft the manuscript. ABA participated in the design of the study and helped to draft the manuscript. CVP participated in the design of the study, the analysis of the data and helped to draft the manuscript. All authors read and approved the final manuscript.

## Pre-publication history

The pre-publication history for this paper can be accessed here:

http://www.biomedcentral.com/1472-6963/12/235/prepub
